# 

**Published:** 1857-03

**Authors:** 


NORTH AMERICAN

### MEDICO-CHIRURGICAL REVIEW.

NO. 2.—MARCH, 1857.

---

### CONTENTS.

### ANALYTICAL AND CRITICAL REVIEWS.

PAGE

ART. I.—1. The History, Diagnosis, and Treatment of the Fevers of the United States. By ELISHA BARTLETT, M.D., late Professor of Materia Medica and Medical Jurisprudence in the College of Physicians and Surgeons of the University of the State of New York, &c. &c. Fourth Edition, revised by ALONZO CLARK, M.D., &c. &c., . . . . . . . . . 161

2. Diseases of the Circulatory System. Division—Fevers—from Elements of Medecine. By SAMUEL H. DICKSON, M.D., LL.D., Professor of the Institutes and Practice of Physic in the Medical College of the State of South Carolina, . . . 161

ART. II.—1. A Treatise on the Diseases of the Breast and Mammary Region. By A. VELPEAU, Professeur à la Faculté de Médecine de Paris, &c. &c. Translated by MITCHELL HENRY, Fellow by examinatian of the Royal College of Surgeons, &c. &c., . . . . . . . . . . . 193

2. A Treatise on Cancer of the Breast and of the Mammary Region. By A. VELPEAU, Member, &c. Translated by W. MARSDEN, M.D., Surgeon to the Royal Free and Cancer Hospital, . . . . . . . . . . . . . . 193

ART. III.—Des Anévrysmes et de leur Traitement. Par PAUL BROCA, Agrégé à la Faculté de Médecine de Paris, &c. &c., . . . 211

ART. IV.—The Causes and Prevention of Yellow Fever at New Orleans and other cities in America. By E. H. BARTON, A.M., M.D., Chairman of the Sanitary Committee; late President of the Louisiana State Medical Society, &c. &c. Third Edition, with the addition of upwards of seventy pages of Prefatory Remarks, and a Supplement, . . . . . . . . . 241

ART. V.—An Exposition of the Signs and Symptoms of Pregnancy; with some other Papers on Subjects connected with Midwifery. By W. F. MONTGOMERY, M.D., &c. &c. Second Edition, . . . 249

ART. VI.—1. The Dissector's Manual of Practical and Surgical Anatomy. By ERASMUS WILSON, F.R.S., &c. Edited by WM. HUNT, M.D., Demonstrator of Anatomy in the University of Pennsylvania. Third American Edition, . . . 254

2. The Practical Anatomist; or the Student's Guide in the Dissecting Room. By J. M. ALLEN, M.D., late Professor of Anatomy in the Medical Department of Pennsylvania College, . . . . . . . . . . . . 254

3. Practical Anatomy; a new Arrangement of the London Dissector, with numerous additions and illustrations. By D. HAYES AGNEW, M.D., Lecturer on Anatomy, . . . 254

ART. VII.—Obstetrics; the Science and Art. By CHARLES D. MEIGS, M.D., Professor of Midwifery and Diseases of Women and Children in Jefferson Medical College, &c. &c., . . . . 258

### ORIGINAL COMMUNICATIONS.

ART. I.—Practical Remarks on Strabismus, with some novel Suggestions respecting the Operation for its Relief. By THOMAS GRAHAM, M.D., F.R.C.S., . . . . . . . . . . . 259

ART. II.—Mortality of Philadelphia for the quarter ending January 1, 1857, and a General Summary for the year 1856. By WILSON JEWELL, M.D., . . . . . . . . . . . 268

ART. III.—Case of Popliteal Aneurism treated by Compression. By T. P. Gibbons, M.D., . . . . . . . . . . . 281

ART. IV.—Case of Hooping-Cough attended with peculiar Symptoms; with Remarks. By CHAS. A. HENTZ, M.D., . . . . . 285

ART. V.—Case of Complete Rupture of the Perineum; Operation; Cure. By B. F. TRABUE, M.D., . . . . . . . . 288

ART. VI.—Reduction of a Femoral Dislocation of Six Months' Standing, by Manipulation. By M. DUPIERRIS, M.D., . . . . 290

ART. VII.—Cases of Inversio Uteri. By P. G. BERTOLET, M.D., . . 294

ART. VIII.—Case of Dislocation of the Head of the Tibia forward. By S. D. GROSS, M D., . . . . . . . . . . 298

ART. IX.—Case of Chronic Peritonitis developed by Pregnancy. By D. B. CLIFFE, M.D., . . . . . . . . . . 299

EDITORS' TABLE, . . . . . . . . . . . . . . 301

EULOGIUM ON ROUX AND REMINISCENCES OF DUPUYTREN, . . . . 312

NECROLOGY, . . . . . . . . . . . . . . . 319

